# Supplementary material for: Development and validation of an artificial intelligence-based pipeline for predicting oral epithelial dysplasia malignant transformation
Source: Commun Med (Lond). 2025 May 20;5:186. doi: 10.1038/s43856-025-00873-z (PMC12092575; doi:10.1038/s43856-025-00873-z)
Supplement: Supplementary file 3 — Description of Additional Supplementary Files [file 43856_2025_873_MOESM3_ESM.docx]

**Description of Additional Supplementary Files**

File name: Supplementary Data 1.

Description: The distribution of dysplasia-epithelium ratios across OED cases based on transformation and grade (source data for Fig. 3).

File name: Supplementary Data 2.

Description: Kaplan-Meier transformation-free survival curves (source data for Fig. 4).
